# PROTAC-Based Protein Degradation as a Promising Strategy for Targeted Therapy in Sarcomas

**DOI:** 10.3390/ijms242216346

**Published:** 2023-11-15

**Authors:** Caterina Mancarella, Andrea Morrione, Katia Scotlandi

**Affiliations:** 1 Laboratory of Experimental Oncology, IRCCS Istituto Ortopedico Rizzoli, 40136 Bologna, Italy; 2 Sbarro Institute for Cancer Research and Molecular Medicine, Center for Biotechnology, Department of Biology, College of Science and Technology, Temple University, Philadelphia, PA 19122, USA; andrea.morrione@temple.edu

**Keywords:** PROTAC, degradation tag, ubiquitination, sarcomas, targeted therapy, fusion genes, BET proteins, BRD9, SMARCA4

## Abstract

Sarcomas are heterogeneous bone and soft tissue cancers representing the second most common tumor type in children and adolescents. Histology and genetic profiling discovered more than 100 subtypes, which are characterized by peculiar molecular vulnerabilities. However, limited therapeutic options exist beyond standard therapy and clinical benefits from targeted therapies were observed only in a minority of patients with sarcomas. The rarity of these tumors, paucity of actionable mutations, and limitations in the chemical composition of current targeted therapies hindered the use of these approaches in sarcomas. Targeted protein degradation (TPD) is an innovative pharmacological modality to directly alter protein abundance with promising clinical potential in cancer, even for undruggable proteins. TPD is based on the use of small molecules called degraders or proteolysis-targeting chimeras (PROTACs), which trigger ubiquitin-dependent degradation of protein of interest. In this review, we will discuss major features of PROTAC and PROTAC-derived genetic systems for target validation and cancer treatment and focus on the potential of these approaches to overcome major issues connected to targeted therapies in sarcomas, including drug resistance, target specificity, and undruggable targets. A deeper understanding of these strategies might provide new fuel to drive molecular and personalized medicine to sarcomas.

## 1. Introduction

Sarcomas encompass a group of tumors of mesenchymal origin, affecting bone and soft tissue. As reported by the World Health Organization (WHO) 2020 Classification of Tumors, sarcomas include more than 100 histological subtypes [1,2]. Approximately 75% of sarcomas arise from soft tissues, about 10% are bone sarcomas, and gastrointestinal stromal tumors (GISTs) represent the remaining 15% [3]. Sarcomas are relatively rare, accounting for 1% of all malignancies in adults, but represent up to 15% of pediatric tumors [4,5]. In the era of molecular medicine, standard of care for local and advanced sarcomas is currently surgery with either neoadjuvant or adjuvant chemotherapy and radiotherapy. The current treatment regimen improved survival rate up to 60% but many patients show mild to low response to chemotherapeutics, requiring higher doses and longer treatment periods [6,7]. In addition, sarcoma recurrence is extremely high, with high incidence of distant metastases, particularly to lungs [8]. Molecular breakthroughs in genomic sequencing techniques coupled with advances in experimental models have improved the deciphering of biological mechanisms underlying onset and progression of different types of sarcomas, resulting in the identification of reliable markers of diagnosis and prognosis [9]. However, these approaches have generated only partial benefits in the development of novel targeted therapies and personalized treatment for sarcoma patients. Excellent reviews have recently discussed sarcoma patients’ response to targeted therapies [4,5,7]. Major factors limiting the efficacy of targeted therapies in sarcoma include (i.) the development of primary and/or acquired resistance, with absence of alternative therapies to overcome the resistance; (ii.) rarity of sarcoma, which limits the possibility to execute subtype- and biomarker-specific trials; (iii.) rarity of actionable mutations in sarcomas; (iv.) limitations in the chemical composition of available inhibitors. Most of the existing molecular targeted therapies act via a mechanism of action based on occupancy, requiring high dosing, high binding affinity, and limited applicability for proteins with nonenzymatic functions, proteins lacking hydrophobic pockets, and proteins with high affinity for substrates [10,11]. Targeted protein degradation (TPD) is emerging as a new molecular tool for blocking oncoproteins in cancer [10]. TPD is based on the use of molecules called degraders, including hetero-bifunctional proteolysis-targeting chimeras (PROTACs) and nonchimeric molecular glues [10,11]. Degraders induce proximity between a recruited E3 ligase and a target protein, mediating the degradation of the entire protein of interest (POI) through the ubiquitination machinery [10,11]. In addition, TPD approaches aim to leverage drug development and the discovery of chemical probes. In recent years, experimental procedures based on engineered systems for ligand-induced protein degradation have been developed and can be used for target validation before the development of a degrader [12].

This review will discuss PROTAC-based protein degradation approaches and focus on how the unique features of TPD may address key challenges in sarcoma treatment. We will also discuss sarcoma subtypes-specific genetic vulnerabilities, which can be exposed to PROTAC-based protein degradation and might represent novel therapeutic opportunities toward precision medicine.

## 2. Fundamentals of Targeted Protein Degradation

TPD relies on the ubiquitin–proteasome system (UPS), a proteolytic mechanism crucial in regulating protein stability and homeostasis as well as trafficking and signaling of receptor tyrosine kinases (RTK) in eukaryotic cells [13,14].

UPS controls fast and efficient elimination of unfolded or misfolded proteins [15], thereby orchestrating crucial cellular functions, including cell cycle control [16,17] and cell fate determination [18]. Accordingly, dysregulation on the UPS is associated with aging as well as pathologic conditions, including neurodegenerative diseases and cancer [19,20,21]. Major experts in the field have previously reviewed the biochemical mechanisms of UPS components and interactors [22]. Briefly, ubiquitination is a three-step cascade-reversible process which involves ubiquitin-activating enzymes (E1), ubiquitin-conjugating enzymes (E2), ubiquitin-protein ligases (E3), deubiquitinating enzymes, and the 26S proteasome. Ubiquitin is a small 8-kDa protein that tags the target protein for degradation but also interactions, conformation, and localization [14]. The ubiquitination cascade occurs as follows: E1 activates ubiquitin in a ATP/Mg2^+^-dependent manner; E2 receives activated ubiquitin from E1 and prepares ubiquitin for conjugation; E3 ligases control substrate specificity, recognizing degradation signals on the target protein, and coordinate ubiquitin transfer to substrate proteins [23]. Extension of ubiquitin chains (polyubiquitination) to a single lysine residue, Lys48, generally confers the pattern for degradation by the 26S proteasome [23]. In the proteasome, the 19S regulatory particle recognizes polyubiquitinated proteins, unfolds the substrates, and translocates them to the 20S catalytic particle; in the 20S chamber, proteins are deubiquitinated and degraded to peptides [22,23].

**Figure 1:**
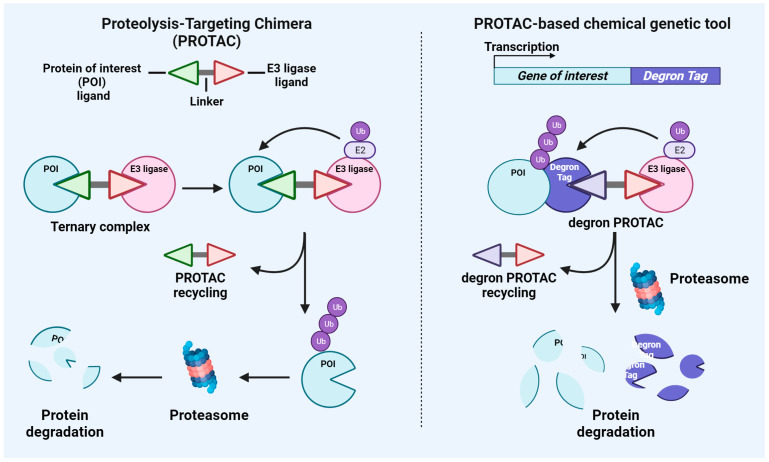
Schematic representation of the mechanisms of action of PROTAC and PROTAC-based genetic tools. The (**left panel**) depicts a schematic of PROTAC structure and its capability to induce protein target degradation through the recruitment of the cellular ubiquitin–proteasome system. The (**right panel**) shows the experimental strategy for genetically fusing proteins of interest with a degron tag susceptible to the binding of specific degron PROTACs which induce proteasome-mediated protein degradation.

TPD takes advantage of this cellular degradation system by recruiting E3 ligases for the degradation of critical POIs. Here, we will focus on a major class of TPDs which are hetero-bifunctional PROTAC and PROTAC-derived experimental systems. However, it must be highlighted that another therapeutic class of degrader is represented by molecular glues, which are not hetero-bifunctional but still enhance the protein–protein interaction between a ligase and a potential substrate to facilitate the ubiquitylation of a POI [24]. A schematic representation of the major features of PROTAC and PROTAC-based genetic tools, which will be discussed below, is shown in Figure 1.

### 2.1. PROTAC

PROTAC is a hetero-bifunctional molecule consisting of three parts: the target protein ligand, the linker, and the E3 ligase ligand. As shown in Figure 1, PROTACs are based on the intracellular UPS system to degrade target proteins of interest in a four-step process: 1. The PROTAC recruits the target protein and the E3 ubiquitin ligase, forming a ternary complex; 2. The target protein is ubiquitinated by the E3 ligase; 3. The ubiquitinated protein is specifically recognized and degraded by intracellular proteasomes; 4. The degrader is released and reactivated in the cycle [10,11,25]. The first chimeric molecule, named PROTAC-1, was described in 2001 by Sakamoto et al. PROTAC-1 recruits the target protein methionine aminopeptidase-2 (MetAP-2) to the ubiquitin ligase Skp1-Cullin-F box complex containing Hrt1 (SCF) [26]. From a structural point of view, PROTAC-1 was composed of two domains: one domain contains the IκBα phosphopeptide recognized by the F-box protein β-TRCP, binding to SCF, and other domain is composed of the angiogenesis inhibitor ovalicin, binding to MetAP-2 [26]. The authors demonstrated that PROTAC-1 mediated the ubiquitination of MetAP-2 and activated MetAP-2 degradation through the intracellular UPS in Xenopus egg extracts [26]. Thus, the authors proposed for the first time that PROTACs could be helpful experimental tools for the manipulation of cellular phenotype and valuable therapeutic agents for degrading disease-promoting proteins [26]. A recent article from Weng et al. provides an updated database collecting all the information from the literature concerning PROTACs [27]. To date, 3270 PROTACs have been described in the literature. Of those, 1095 were tested in cellular models; 2073 were tested via western blotting for target validation; and 1070 were tested for binding affinity data to target proteins, E3 ligases, and formation of ternary complexes [27]. Of note is that the number of target proteins increased from 147 in 2021 [28] to 280 as of today [27], demonstrating the great interest of the research community in this field and the capability of PROTACs to extend the “druggable” space [10]. From the database created by Weng et al., it appears that most of the PROTACs available in the literature target androgen receptor (AR), followed by the bromodomain and extraterminal (BET) domain protein BRD4 and estrogen receptor (ER). However, it must be highlighted that, given its chimeric nature, the development of a PROTAC presents several potential challenges. Below, we will discuss major features of each of the three components of a PROTAC, highlighting specific advantages and issues. First, the moieties targeting the POI are given by the target ligand. To this end, it is not surprising that the most common PROTACs described in the literature are based on the use of previously used small molecule inhibitors, including BET protein inhibitor JQ1 [29] or AR inhibitor enzalutamide [30]. In most cases, the identification of a small molecule targeting the POI represents a challenging and time-consuming task, particularly for those proteins which lack a well-defined active site. However, PROTACs bind to a POI and modulate its function without the need of occupancy-based ligands. A small molecule ligand can target any part of a protein, including functionally inactive domains, and still act as an effective PROTAC. Second, the identification of the E3 ligase optimal for the degradation of the POI is another crucial aspect during the development of a PROTAC. Although there are more than 600 E3 ligases encoded by human genome, only a few have been successfully used as PROTACs, including cereblon (CRBN), von Hippel–Lindau (VHL), MDM2, and cIAP. Of the existing PROTACs, almost 63% of them incorporate the CRBN E3 ligase [31], while 31% use VHL, indicating the predominance of these E3 ligases as moieties. This is due to the ubiquitous expression of those molecules across tissue types and the existence of well-characterized ligands of these two E3 ligases [10]. CRBN is a substrate adapter for the Cullin-RING E3 ubiquitin ligase complex. Ligands recruiting CRBN include thalidomide and its derivatives (IMiDs), such as pomalidomide and lenalidomide [32]. IMiDs functionally act by modulating the selection of protein substrates for ubiquitination and degradation [33]. Functional substitutes of thalidomide have been described, including the cyclic imide peptide FcQ [34]. Treatment with thalidomide-based BRD4 degraders PROTAC dBET6 or JQ1-FcQ potently degraded BRD4 protein. Of note is that treatment with MLN4924, which inhibits the activity of Cullin-RING E3 ligases, rescued the degradation of BRD4 [34]. VHL protein is a substrate of the recognition subunit of a Cullin 2-RING E3 ligase complex [35]. Ligands of the E3 ligase VHL were originally identified based on a specific peptide of hypoxia-inducible factor 1 subunit-α (HIF1α) [36,37]. Starting from those peptide ligands, several chemical compounds have been developed to selectively bind VHL with distinct applications, including PROTACs. Different studies have demonstrated the utility of VHL ligands, including VH298 and VH032 in PROTACs targeting kinases, such as SGK3 [38], BET proteins [29], or VHL itself [39]. Other E3 ligases more commonly used for PROTAC development include cIAP1, IAP, XIAP, and MDM2, while less common is the use of E3 ligases, such as DCAF15, DCAF16, FEM1B, RNF4, AhR, DCAF11, RNF114 [27]. Third, the degradation efficiency of a PROTAC also relies on a suitable linker which allows an efficient ternary complex formation and POI ubiquitination [40]. Recent research has demonstrated that a constant warhead can be used to target just one particular isoform of a protein family by altering the PROTAC linker design and encouraging differential orientation of a single recruited E3 ligase [40]. The functionality of both the E3 ligand and POI ligand predetermines the type of chemistry for linkers, either rigid or flexible [41]. PEG and alkyl are the two most typical linker motifs, and modifications to the chain length and composition affect the physicochemical characteristics and the activity of these degraders [42].

### 2.2. PROTAC-Based Chemical Genetic Tool

PROTAC-based tools have been developed as novel genetic suppression strategies for target validation in vitro [12]. As shown in Figure 1, these systems are based on the use of specific tags, named degron, which are genetically fused to the POI and render the POI sensitive to protein degradation given by treatments with degrader drugs, including PROTACs and molecular glues. Thus, treatment of cell lines expressing the degron-tagged POI with the cognate degron drug induces degradation of the POI. Compared to other gene suppression approaches, such as short hairpin RNA (shRNA) and CRISPR/Cas9, chemical genetic tools might limit off-targets effects, allowing a rapid mechanism of action, reversible perturbation of the target protein and the possibility to measure the extent of target protein inhibition. Below, we will discuss major features of the two systems based on specific PROTACs, including the degradation tag (dTAG) system and the HaloTag system. However, it must be highlighted that other systems exist and are based on the use of molecular glues, including the auxin-inducible degron (AID), IKZF3d degron, and SMASh [12]. From a technical standpoint, the dTAG and HaloTag systems are based on the use of lentiviral vectors for stable integration of the degron tag into the genome. In the plasmid design, the degron tag is fused in-frame to either the N-terminus or C-terminus of the POI.

The dTAG system is based on the ligand AP1867, which selectively binds the mutant variants of FKBP12 protein, FKBP12^F36V^, but not its wild type form [43,44,45,46]. dTAG-related PROTACs include dTAG-13, which recruits CRBN, and dTAG^V^-1, recruiting VHL. The dTAG-PROTACs mediate the degradation of FKBP12^F36V^-fused POI [43,44]. There are differences between dTAG-13 and dTAG^V^-1 as in fact, the two PROTACs can degrade different targets. The FKBP12^F36V^-EWS::FLI1 fusion protein was prone to degradation mediated by dTAG^V^-1 but not dTAG-13, evidencing the necessity of comparative analysis between VHL-recruiting and CRBN-recruiting dTAG PROTACs [43]. Different studies have demonstrated the applicability of both dTAG-13 and dTAG^V^-1 PROTACs for in vivo studies [43,47]. For instance, mice with homozygous knock-in of the dTAG onto *CDK5* and *CDK2* treated with dTAG-13 displayed a substantial degradation of the two proteins in all organs except the brain, possibly due to the inability of dTAG-13 to diffuse through the blood–brain barrier [47]. A direct comparison between dTAG-13 and dTAG^V^-1 indicated that dTAG^V^-1 demonstrated improved pharmacokinetic properties compared to dTAG-13, including longer half-life and greater bioavailability, via intraperitoneal administration [43]. dTAG^V^-1 determined prolonged degradation as compared to dTAG-13, as in fact, degradation was still evident 24 h after the final administration, as demonstrated in vivo after tail vein injection of bioluminescent MV4;11 luc-FKBP12^F36V^ cells [43].

HaloTags are modified haloalkane dehalogenase designed to covalently bind to synthetic ligands [48]. The HaloTag-related PROTAC, named HaloPROTAC, includes a chloroalkane moiety, which binds the HaloTag and a moiety which recruits VHL through either a hydroxyproline derivative (HaloPROTAC3) [49] or VH298 (HaloPROTAC-E) [50]. A head-to-head comparison of HaloPROTAC3 and HaloPROTAC-E was recently performed to evaluate dose- and time- dependent degradation of Halo-tagged Class III PI3K VPS34 [50]. HaloPROTAC-E showed greater potency in inducing Halo-VPS34 degradation compared to HaloPROTAC3 [50]. Accordingly, 10 nM of HaloPROTAC-E induced a 15% higher degradation of Halo-VPS34 than HaloPROTAC3 used at the same concentration after 24 h of treatment in vitro [50]. Moreover, at 1–4 h, around 75% degradation of Halo-VPS34 was observed with HaloPROTAC-E, compared to 50% with HaloPROTAC3 [50]. However, additional studies are necessary to clearly establish HaloPROTACs different properties, taking into account POI and their localization. HaloPROTAC3 was shown to effectively work in mice [51,52], as in fact, mice expressing HaloTag-fused POI (PNPLA3 protein) with HaloPROTAC3 showed decreased protein but not transcript levels of PNPLA3 [51].

Recently, Bondeson et al. performed a systematic profiling of different degron tag technologies across 16 different protein targets [12]. Interestingly, the authors observed that target degradation was highly dependent on the employed degron tag, the design of the lentiviral construct, and the POI itself. Of note is that none of the considered degron tags caused efficient degradation of all targets, but at least one optimal degron tag existed for each POI [12]. Despite the major advantages of degron tag technologies, these techniques are not without limitations. For instance, insufficient degradation can associate with wrong tag/fusion design or elevated baseline expression levels in cells. As a consequence, the tagged protein does not phenocopy the inactivation of the target gene. Alternatively, the interference of the tag with POI activity or low expression of tagged POI can reduce the efficiency of the system. In this case, the tagged protein does not phenocopy the functions of the wild type POI. A functional degron tag is optimal when the tagged POI phenocopies both the inactivation of the target gene upon degrader treatment and the expression of the endogenous gene [12].

A recent systematic study has investigated the impact of POI localization, comparing HaloTag vs. dTAG degradation efficiency in the cytoplasm, nucleus, Golgi (facing either cytoplasm or lumen), endoplasmic reticulum, outer mitochondrial membrane, lysosome, and peroxisome [46] using VHL- or CRBN-recruiting PROTACs [46]. Matching the ligands of the POI and the E3 ligase during PROTAC development requires an understanding of how the target protein’s subcellular localization and the type of the E3 ligase to recruit affect PROTAC-mediated degradation [46]. The authors show that different PROTACs specifically degraded proteins localized in the cytoplasm, nucleus, endoplasmic reticulum, outer mitochondrial membrane, lysosome, and peroxisome [46]. However, proteins localized in the inner lumen of the Golgi were resistant to degradation [46]. In particular, the Halo/VHL-recruiting PROTAC—HaloPROTAC-E—degraded Halo-tagged proteins that were localized to all of the subcellular compartments mentioned above, with the exception of the Golgi lumen. Contrarily, only FKBP12^F36V^ proteins that were localized in the cytoplasm, nucleus, or endoplasmic reticulum were effectively degraded by the FKBP12^F36V^/VHL-recruiting PROTAC dTAGV-1. On the other hand, FKBP12^F36V^-tagged proteins localized in the cytoplasm, nucleus, outer mitochondrial membrane, and endoplasmic reticulum were found to be degraded by dTAG-13 CRBN-recruiting PROTAC [46].

## 3. Can Targeted Protein Degradation Overcome Major Challenges in Cancer Treatment?

Advances in CRISPR-based screening technologies coupled with cutting-edge next-generation sequencing approaches have enhanced the identification of numerous novel potential therapeutic targets in human pathologies, particularly in cancer. However, solid validation of targets is required before investing in drug discovery. Major difficulties for target validation include kinetic differences between genetic manipulation and pharmacological inhibition. PROTAC technologies have been developed to induce the degradation of oncoproteins, such as ER, AR, BCR-ABL, BRD4, RIPK2, MET, EGFR, p38, BTK, ERRα, and MAPK [53], and almost 26 PROTACs have entered clinical trials [10]. PROTAC degraders can be orally available, can diffuse broadly in tissues, and are also active in the central nervous system. Accordingly, some PROTACs can diffuse through the blood–brain barrier in spite of their high molecular weights (often higher than 1 KDa) [54,55,56], opening the possibility of using these molecules for the treatment of neural diseases, including brain tumors such as glioblastoma [57] and disorders like Parkinson’s disease [54] and Alzheimer’s disease [58]. Many previous reviews have nicely described the relevance of TPD in cancer [10,11,59,60]. This section will highlight some examples of preclinical and clinical settings regarding TPD features to broaden *druggable* cancer targets, to address drug resistance, and to enhance target specificity as well as concerns regarding off-tumor/off-target toxicity and emerging mechanisms of resistance to TPD. Table 1 summarizes data discussed in the review and presents tumor types and target proteins to which TPD-based approaches have been used.

### 3.1. Targeted Protein Degradation and Undruggable Cancer Targets

TPD approaches are now drawing interest as next-generation drugs to target currently *undruggable* proteins [83]. Schneider and colleagues have recently presented a systematic approach to assess the PROTAC-tractability (PROTACtability) of protein targets in the proteome using different criteria based on information and data from public databases [84]. The criteria included protein cellular location, evidence of ubiquitylation residues, protein half-life, presence of one or more small molecule ligands, previously reported targets in the PROTAC literature, and PROTAC molecules in clinical development [84]. The findings of this study showed that a total of 1336 targets would currently be regarded as PROTACtable among the currently known 19,498 protein-coding genes [84]. Of those, 1067 targets were assigned to a “discovery opportunity category”, highlighting the potential benefit of TPD for future drugs development [84]. In support of the utility of such a prediction system towards the PROTACtability of unexplored molecules, Bensimons et al. have recently demonstrated that five transmembrane solute carrier (SLC) proteins, included in the list of PROTACtable proteins, are indeed degradable with a PROTAC approach [84,85]. SLCs represent the largest class of transporters in the human genome [85]. Recent evidence demonstrates that some SLCs play critical roles in tumorigenesis, indicating that SLCs can be interesting targets for drug development in cancer [85]. However, their numerous transmembrane domains and hydrophobicity severely contributed to the absence of specific therapeutic agents targeting these molecules [84,85]. The authors analyzed the possible use of TPD for SLCs by using the dTAG system. In cellular models, different SLCs were expressed as fusions to the mutated FKBP domain, and then protein degradation was assessed upon treatment with dTAG^V^-1 or dTAG-13. dTAGed-SLCs indicated that different SLCs could be recruited by the proteolytic machinery, thereby responsive to proteasome-mediated TPD. The authors then selected SLC9A1 for the subsequent development of a novel SLC degrader named d9A-2 [85]. d9A-2 is composed using a modified version of a previously reported ligand of SLC9A1 linked to the phthalimide-based CRBN ligand [85]. This degrader displayed potent cytotoxic activity in a variety of tumor cell lines, demonstrating its potentiality for preclinical development [85].

Other previously considered “*undruggable*” proteins include transcription factors and RNA-binding proteins. A crucial advantage of the PROTAC system over small molecules inhibitors is that a weak POI ligand can still act as a potent PROTAC. This is a crucial advantage in the development of PROTACs targeting transcription factors, which remain very difficult to target using occupancy-based small molecules lacking *ligandable* sites [86]. Lee et al. developed MTX-23, a PROTAC targeting the “*undruggable*” androgen receptor (AR) splice variant ARV7It, from ligands that moderately inhibit the AR DNA-binding domain [61]. Other transcription factors lacking specific inhibitors include the zinc finger (IKZF) transcription factors Aiolos (IKZF3) and Ikaros (IKZF1), two validated drug targets of different cancers [82]. As recently shown, the CRBN-based PROTAC MS40 induce simultaneous degradation of IKZF1 and IKZF3, suppressing tumor cell growth in vitro and in vivo [82]. Another approach to the degradation of transcription factors is to link a DNA oligonucleotide to an E3 ligase ligand to selectively degrade the transcription factor of interest [87]. Two PROTACs recruiting VHL and targeting transcription factors have been recently described including E2F-PROTAC (dE2F) and NF-κB-PROTAC (dNF-κB), which efficiently degrade E2F1 and p65 proteins, respectively, and show antiproliferative effects in cancer cells [87]. Similarly, a chimeric oligo sequence connected to an E3-ligase-recruiting ligand makes up the molecule known as transcription factor targeting chimeras (TRAFTACs), which was created by Samarasinghe et al. [86]. The chimeric oligo was composed of double-stranded DNA (dsDNA) that can bind a transcription factor of interest and an RNA that can bind CRISPR/Cas9 [86]. The dsDNA binds the transcription factor, whereas the CRISPR-RNA binds to an ectopically expressed dCas9-Halotag7 fusion protein (dCas9HT7). Incubation with a HaloPROTAC then attracts the VHL-E3 ligase to the DNA-bound transcription factor, causing ubiquitination and proteasomal degradation of the POI [86]. By customizing the DNA sequence, the authors demonstrated the capability of TRAFTACs to target different oncogenic transcription factors, including NF-κB and the T-Box transcriptiona factor T in vitro and in zebrafish models [86].

Similarly, RNA-binding proteins are difficult to target due to their structural features, including variable RNA binding pockets and high homology between RNA-binding domains. Still, experimental evidence indicates that RNA-binding proteins might be susceptible to protein degradation. To this end, Worner et al. have recently shown that the knock-in of the dTAG to the locus of the RNA-binding-protein *Trim71* coding gene enables inducible and rapid Trim71 protein degradation in vitro [88]. Intriguingly, the application of the dTAG system brought to light a warning when interpreting the outcomes of studies on RNA-binding proteins that involved loss of function. Although the levels of AGO2 protein are unaffected in *Trim71* knockout cells, inducible degradation of Trim71 causes the expected rapid increase of AGO2 and subsequent return to basal levels, demonstrating that *Trim71* knockout has secondary effects that might counteract its direct effects on *AGO2* mRNA [88]. In 2021, Ghidini and colleagues developed a new type of PROTAC named RNA-PROTAC [80]. The RNA-PROTAC uses a short oligoribonucleotide as a ligand for the POI. Specifically, the authors developed the compound ORN3P1, which includes the key partial sequence (AGGAGAU) of *pre-let-7* that binds to Lin28 as the POI ligand together with a peptide ligand for VHL E3 ligase [80]. As shown, ORN3P1 selectively bound to Lin28 in vitro, inducing Lin28A degradation in a ubiquitin-dependent fashion in two cancer cell lines [80]. Recent data demonstrate that the oncogenic RNA-binding protein IGF2BP3 is a substrate of different E3 ligases including MKRN2 [89], entailing the development of PROTAC as a novel approach to target this molecule. Overall, these results indicate that the development of PROTAC against RNA-binding proteins could be feasible.

### 3.2. Targeted Protein Degradation and Drug Resistance

Resistance mechanisms to targeted therapies in cancer include overexpression, point mutations, kinase-independent (scaffolding) functions of the POI as well as activation of compensatory pathways. Due to their mechanisms of action, TPD approaches show promise for overcoming those obstacles to effective cancer therapy.

In 2018, Salami et al. performed a side-by-side comparison between the AR inhibitor enzalutamide and its PROTAC counterpart, named ARCC-4, using prostate cancer cellular models of drug resistance [30]. ARCC-4 PROTAC presents the AR inhibitor enzalutamide fused to a VHL E3 ligase ligand [30]. ARCC-4 induced selective degradation of AR via the proteasome in multiple cancer cell lines and was more effective than enzalutamide in inducing apoptosis, inhibiting cell proliferation and blocking AR signaling in AR-overexpressing prostate cancer cells [30]. Interestingly, ARCC-4 was not effective when administered to AR-negative PC3 prostate cancer cells or AR-positive cancer cells transfected with *VHL* siRNA [30]. ARCC-4 exemplifies how protein degradation can address drug resistance in conditions of POI amplification or expression of clinically relevant AR mutants. For instance, the AR mutation F876L mediates enzalutamide resistance by promoting enzalutamide action as an agonist. ARCC-4 treatment of prostate cells engineered to overexpress the AR-F876L mutant did not increase PSA levels as compared to treatment with enzalutamide [30].

Bruton’s tyrosine kinase (BTK) targeting using irreversible inhibitors, such as ibrutinib, represents a transformative option for treatment of patients with chronic lymphocytic leukemia (CLL) and other B-cell malignancies. However, resistance to these inhibitors has been reported, and it is mostly associated with mutations in the *BTK* gene, preventing the irreversible binding of inhibitors [77,90]. The most common BTK mutation is the substitution of the cysteine 481 residue with serine (C481S). To this end, BTK PROTACs have been developed to overcome resistance [77,90]. The CRBN-recruiting PROTAC UBX-382 efficiently degrades and strongly represses the phosphorylation of wild type and different BTK mutants, with the only exception of T474I [77]. In addition, UBX-382 inhibited cell proliferation in both ibrutinib-sensitive parental TMD-8 and wild type cells as well as in ibrutinib-resistant TMD-8 cells overexpressing mutant C481S BTK, indicating that UBX-382 can overcome drug resistance in C481S mutants [77].

Alabi et al. developed SJF-0628 PROTAC, by linking vemurafenib to a VHL E3 ligase and using a rigid linker as a strategy to target mutant-BRAF-driven tumors [53]. SJF-0628 exhibited a significant antitumor effect in vitro and in vivo [53]. Interestingly, SJF-0628 determined efficient degradation of mutant BRAF while sparing wild type BRAF [53]. In contrast to mutant BRAF, wild type BRAF only weakly interacts with the E3 ligase complex in cells, resulting in limited ubiquitination and degradation. Thus, these data suggested mutant selectivity of the PROTAC [53]. In addition, the authors successfully targeted vemurafenib-resistant BRAF mutations. These included mutants associated with both intrinsic and acquired (p61 V600E) resistance to vemurafenib [53]. PROTAC holds the potential to be used when kinase activity is not the only function of the target protein [79]. This is the case of the oncogenic fusion protein BCR-ABL1 in chronic myeloid leukemia (CML) [79]. The use of tyrosine kinase inhibitors (TKI) such as imatinib to block BCR-ABL1 kinase activity has significantly improved prognosis for patients [79]. However, drug resistance can occur, and it is associated with kinase-independent and scaffolding-dependent BCR-ABL1 functions, which modulate the interaction with Shc and subsequent recruitment of GRB2 to induce survival mechanisms [79]. Degraders of BCR-ABL1 can play a role in overcoming resistance to TKIs [78,79]. The BCR-ABL1 PROTAC DMP11 significantly induced protein degradation of BCR-ABL1 as well as downstream SRC protein in vitro, thereby inhibiting cell viability of both imatinib-sensitive (K562) and imatinib-resistant (KA) chronic myeloid leukemia cell lines [78]. DMP11 displayed in vivo therapeutic efficacy on imatinib-resistant-KA-induced orthotopic animal models. In this system, DMP11-treated mice showed decreased tumor progression and increased survival compared to the control group [78]. These data are consistent with previous reports demonstrating that BCR-ABL1-dependent drug resistance driven by scaffolding mechanisms may be addressed by a strategy that combines BCR-ABL1 kinase inhibition and protein degradation. The BCR-ABL1 PROTAC GMB-475 developed by Crews’s Lab, comprised of an allosteric ABL1 inhibitor GNF-5 and a VHL-recruiting ligand, induced in vitro proteasomal degradation and inhibited cell proliferation of both wild type BCR-ABL1 as well as clinically relevant T315I BCR-ABL1 kinase domain mutants [79]. Reverse-phase protein array analysis revealed that activation of downstream oncogenic SHP2, GAB2, and Shc pathways is linked to kinase-independent mechanisms mediated by BCR-ABL1 [79]. Of note is that the combined treatment with GMB-475 reduced the IC50 of imatinib in BCR-ABL1–transformed Ba/F3 cells, demonstrating that dual treatment with an active degrader is more effective than a combined treatment with the equivalent allosteric inhibitor [79].

Recently, a PROTAC library was developed to degrade both IGF-1R and Src proteins. Based on the observation that Src activation mediates the resistance to different anti-IGF-1R therapeutics, Manda et al. designed and synthesized IGF-1R/Src dual PROTAC degraders [63]. Using the pomalidomide as a ligand for CRBN E3 ligase in breast cancer cells, CPR3 and CPR4 PROTAC compounds induced IGF-1R and Src degradation, which was associated with decreased cell viability, reduced migration, and invasion and diminished growth in anchorage independency [63].

However, cancer cells can develop resistance to PROTACs, and this is an important issue in the field [91]. The study of Kurimchak et al. demonstrated that the intrinsic elevated expression of drug efflux MDR1 proteins or drug-induced production of MDR1 mediated resistance to PROTACs targeting KRAS or pathway-associated proteins, including FAK, CDK9, BRD4, or MEK [92]. Accordingly, PROTAC-resistant cells were re-sensitized to PROTACs through co-treatment with MDR1 inhibitors or through genetic suppression of the MDR1 encoding gene *ABCB1* [92]. Other reported mechanisms of resistance to PROTAC include loss-of-function mutations in *CRBN* or *VHL* coding genes [91,93,94]. Concerning BET-PROTAC, deletion of the 12 Mbp fragment of the chromosome 3 containing the *CRBN* gene caused resistance to CRBN-recruiting PROTACs [91,94]. In parallel, mutations of Cullin 2 (*CUL2*) determined the resistance to PROTACs recruiting VHL E3 ligase [91,94].

### 3.3. Targeted Protein Degradation and Target Specificity

Specificity is one major limitation in the effective clinical use of anticancer compounds, particularly small molecule inhibitors. The inhibitors of the BET family of proteins are one example. BET proteins, including BRD2, BRD3, BRD4, and BRDT, act as epigenetic regulators by recognizing acetylated lysine residues in nucleosomal histones, thereby activating transcription [95]. Alterations in the expression or functions of BET proteins, particularly BRD4, are associated with pathologic conditions, including human cancers [95]. To displace BET proteins and their transcriptional regulatory complex from chromatin, various small molecules inhibitors have been developed to bind the pocket of bromodomains and disrupt the interaction with histones [96]. BET inhibitors TEN-010, PLX51107, I-BET762, or OTX015, are all similar in their structure to the widely used JQ1 and have also been tested in clinical trials [95]. Different PROTAC compounds based on BET inhibitors have been recently developed. In 2015, Ciulli’s Lab published results on a novel PROTAC named MZ1, which tethers JQ1 to a ligand for the E3 ubiquitin ligase VHL [29]. Interestingly, MZ1 exhibited selective removal of BRD4 over BRD2 and BRD3, which was quite surprising considering that JQ1 is a pan-BET inhibitor [29]. Accordingly, the authors demonstrated that MZ1, contrary to JQ1, differentially affected mRNA expression of a selection of BET-related genes. Overall, gene expression changes upon MZ1 treatment resembled those associated with siRNA-induced loss of *BRD4* [29] indicating that a more BRD4-selective pharmacological profile can be achieved with MZ1 as compared to pan-selective inhibitor JQ1. PROTAC selectivity is not completely understood, but the hypothesis is that it is governed by a combination of molecular recognition, availability of targets, protein turnover rates, and ternary complex formation. Accordingly, even highly promiscuous ligands can work as surprisingly selective degraders [97]. MZ1 analogues have also been used to improve tissue selectivity, in order to enhance delivery of the PROTAC into cancer cells. To this end, antibody–PROTAC conjugate have been developed as alternative approaches for the selective delivery of broad-spectrum PROTACs into specific cell types [64]. Though BRD4 is the most studied BET protein, several reports suggest that BRD2 and BRD3 may also be crucial in the dysregulation of oncogenes in cancer [95]. Notably, a novel PROTAC degrader 24 promotes selective degradation of BRD3, BRD4, and BRD4-L, and showed robust antitumor activity both in vitro and in vivo [98]. The observed target selectivity was in part attributed by the authors to differences in protein degradation kinetics [98].

Similarly, STAT proteins represent a family of signal transducers and transcription factors (STAT1-STAT6) frequently dysregulated in cancer and with an important role in cancer progression, particularly for STAT3 and STAT5 [99]. STAT5 inhibitors generally lack selectivity over other STAT proteins. Recent findings indicate that AK-2292 is a potent STAT5 PROTAC degrader [81]. A structure-guided design of optimal STAT5 SH2 domain ligands allowed the synthesis of the AK-2292 compound, which induces CRBN-mediated degradation of STAT5 proteins, but not STAT1, STAT2, STAT3, STAT4, or STAT6 [81]. Accordingly, unbiased proteomics analyses indicated that AK-2292 reduced the levels of STAT5A and STAT5B proteins by >85% and had no significant effect on any other 6000 proteins analyzed [81]. Target specificity was also confirmed via RNA-seq analysis, which indicated that AK-2292 specifically downregulated STAT5-associated genes in cancer cells [81].

One of the major concerns in cancer treatments is host toxicity, as effective PROTACs can also induce protein degradation in normal tissues after systemic administration. Thus, future studies and clinical trials will be important to further characterize safety, efficacy and global impact of PROTACs [62]. As recently reviewed, PROTAC targeting estrogen receptors (ER) including ARV-471 (Arvinas), AC682, and DT2216 [62] are currently in clinical trials. Results from phase I/II trials testing ARV-471 in patients with ER+/HER2- breast cancer indicated the presence of side effects. In particular, during the dose escalation phase, side effects included nausea, fatigue, and vomiting [62]. In the phase II dose expansion, 23% of patients (16 out of 71 patients) developed grade >3 adverse events, including acute respiratory failure [62]. Thus, novel approaches are currently under development to increase PROTAC specificity. Data currently available in literature demonstrate the efficacy of antibody–PROTAC conjugates. Maneiro et al. developed a trastuzumab–BRD4 degrader conjugate to achieve cell-type-specific BRD4 degradation in HER2+ breast cancer cells [64]. This molecule, named Ab-PROTAC 3, was synthesized by conjugating the MZ1 analogue PROTAC 1 to the monoclonal antibody trastuzumab [64]. Ab-PROTAC 3 selectively degraded BRD4 in HER2+ cells but not in HER2– cells, as shown in vitro [64]. On the contrary, PROTAC 1 treatment determined BRD4 degradation in both HER2-positive and HER2-negative breast cancer cells, providing the proof of concept for selective HER2-mediated PROTAC delivery and BRD4 degradation by Ab-PROTAC 3 [64]. Another approach to limit off-target toxicity relies on the development of PROTACs responding to exogenous stimuli, including hydrogen-peroxide-inducible [66] or light-controllable PROTACs (photo-activable nano-PROTAC) [100], and bio-orthogonal on-target activated PROTAC prodrugs [65]. In these systems, a PROTAC precursor or PROTAC prodrug is administered to tumor cells. The hydrogen-peroxide-inducible PROTACs has an H_2_O_2_-sensitive group and H_2_O_2_-inducible PROTAC precursors 2/5, which is activated by endogenous H_2_O_2_ in cancer cells to release the active PROTACs 1/4 and effectively degrade targeted proteins [66]. On the contrary, normal cells, characterized by low H_2_O_2_ concentration, will remain unaffected [66]. Light-controllable PROTACs use either photo-switch or photo-cage approaches, which are commonly used in photodynamic therapy [100]. Photo PROTACs use UVA light to activate or inactivate the PROTAC and control the spatio-temporal degradation of the protein of interest [100]. These PROTACs hold a photolabile group sensitive to light. Treatment with UVA activates particular PROTACs, which can induce degradation of the POI. UVA-induced degradation was achieved for different oncogenic proteins including BET proteins, by using caged-dBET1 PROTAC, or ALK, by using caged-dALK [67]. The bio-orthogonal PROTACs enable on-target activation of PROTAC prodrugs and the selective release of PROTACs in cancer cells [65]. In this system, the PROTAC is an inert prodrug since it is caged with a trans-cyclooctene (TCO; TCO-PROTAC) linked to the ubiquitin ligase ligand [65,101]. Cells are co-treated with TCO-PROTAC and a tetrazine (Tz) moiety conjugated with the c(RGDyK) ligand specific for αvβ3 integrin, which is highly expressed on the surface of some cancer cells. Thus, co-treatment activates the TCO–ARV-771 prodrug through a bio-orthogonal click reaction between TCO and Tz exclusively in cancer cells [101]. The authors employed ARV-771, a VHL-based BET degrader [65], and BRD4 degradation was observed in tumor cells but not in normal cells, indicating that this strategy can work as an effective method for causing cancer-selective cell death through the ubiquitin–proteasome pathway [65].

## 4. Perspectives for the Use of Targeted Protein Degradation-Based Strategies in Sarcoma Treatment

At the molecular level, sarcomas have been traditionally classified into two major categories: sarcomas with simple, near-diploid karyotypes and sarcomas with complex karyotypes characterized by multiple genomic aberrations. However, the most recent genome- and epigenetic-wide profiling have highlighted that these two categories do not reflect the elevated clinical and biological heterogeneity present the various known subtypes of sarcomas. In addition, molecular profiling approaches have identified novel reliable diagnostic, prognostic, and potentially therapeutic factors for targeted therapy. In sarcoma, RTK inhibitors and monoclonal antibodies constitute a feasible approach for tumor control. These agents include inhibitors of VEGF, PARP, IGF-1R, CDK4/6, mTOR, and c-MET [4,5,7]. However, a few achievements from the use of biology-guided administration of targeted therapies have been obtained in GISTs and Ewing sarcoma, displaying a 85% response to the PDGFR/KIT inhibitor imatinib, and a 10% response to anti-IGF-1R inhibitors, respectively [102,103]. In parallel, immunotherapy-based strategies, including immune checkpoint inhibitors, oncolytic viruses, and CAR-T cells have shown limited efficacy in initial studies [4,5,7]. This section will cover major molecular mechanisms involved in sarcoma malignancy and current targeted therapies as well as preclinical and clinical results using PROTAC-based approaches, focusing on their specific advantages over traditional inhibitors.

### 4.1. Targeting the Undruggable EWS::FLI1 in Ewing Sarcoma

Ewing sarcoma (EWS) is the second-most common tumor of bone affecting pediatric patients. EWS is characterized by high propensity for metastases, which are the major determinants of patient poor prognosis. Standard of care—consisting of surgery and/or radiotherapy and multiagent chemotherapy—has improved survival for patients with localized tumors (70%), but survival remains dismal for patients with metastatic disease (30%) [4]. The genetics of EWS are characterized by a chromosomal translocation that fuses the gene encoding EWSR1 RNA-binding protein with a member of the ETS family of transcription factors, primarily FLI1 (85–90% of cases), leading to the chimeric protein EWS::FLI1 [4]. Genome-wide sequencing studies demonstrated that EWS has one of the lowest mutation rates among tumors [104], supporting the notion that EWS::FLI1 is the driving oncogene in this tumor. EWS::FLI1 promotes the tumorigenesis and progression of EWS by acting not only as an aberrant transcription factor by recruiting chromatin remodeling complexes, such as the BAF complex, but also as an epigenetic modulator by altering DNA methylation, histone modifications, and alternative splicing [104,105]. Thus, EWS::FLI1 represents a major dependency in EWS, and efforts have been made to target this chimeric protein and/or its critical interactors, as recently reviewed by Flores and colleagues [105]. Those approaches include oligonucleotides targeting *EWS::FLI1*; epigenetic agents, including BET inhibitors; histone demethylase inhibitors; histone deacetylase inhibitors; and agents targeting EWS::FLI1 downstream targets, including the IGF-1R, GLI1, Casein kinase 1 (CK1) [105]. In addition, pharmacological inhibition of EWS::FLI1 interactome to modulate splicing using YK-4-279 and its analog TK-216 represented an excellent strategy for targeting EWS [106]. YK-4-279 disrupts the interaction between EWS::FLI1 and its transcriptional coactivator RNA helicase A (RHA), thereby inhibiting the transcription of target genes and impeding alternative splicing modulation [106,107]. Accordingly, YK-4-279 effectively inhibited in vivo xenograft tumor growth [108]. Results from a phase I trial of TK-216 in combination with the chemotherapeutic agent vincristine indicated that the drug was well tolerated and showed antitumor activity with an overall clinical benefit rate of 64% [109]. Still, as shown in in vitro preclinical studies, resistance to YK-4-279 can occur, and EWS cell lines resistant to this compound display a different transcriptional landscape when compared to sensitive cells [108].

**Figure 2:**
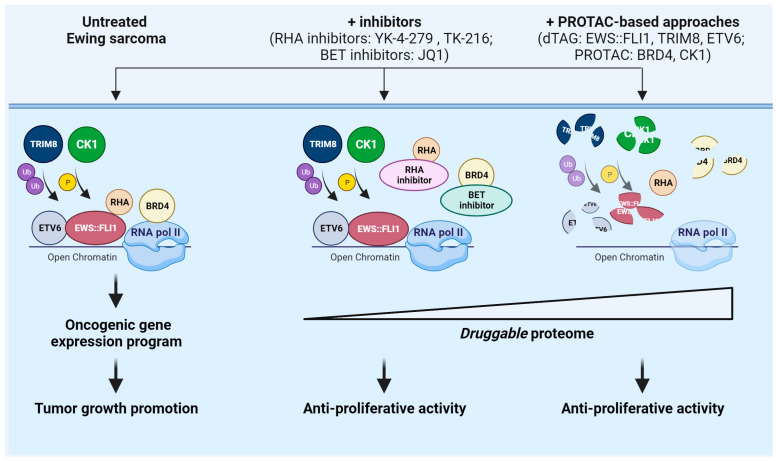
Schematic representation of targeting strategies for EWS::FLI1 and relative interactors using PROTAC-based protein degradation compared to small molecules inhibitors in EWS. On the (**left**), EWS::FLI1 acts in complexes also containing ETV6, BRD4, and RHA, while protein degradation of EWS::FLI1 is regulated by TRIM8 E3 ligase and CK1; in the (**center**), available inhibitors against these mediators include BET inhibitors, which block the BET proteins binding to target genes, and RHA inhibitors, which disrupt the interaction between EWS::FLI1 and RHA; on the (**right**), PROTAC-based approaches including the dTAG system and PROTAC degraders may deplete EWS::FLI1, TRIM8, ETV6, CK1, and BRD4. Biological responses critical for gene transcription and downstream cell proliferation are reported.

Recent evidence supports the notion that targeting EWS::FLI1 for degradation with hetero-bifunctional degraders could be a viable strategy [43]. Using dTAG approaches in EWS cell lines, Nabet et al. demonstrated that FKBP12^F36V^-EWS::FLI1 is specifically susceptible to VHL-recruiting dTAG^V^-1 molecule degradation. Of note, treatment with dTAG^V^-1 led to decreased expression of downstream EWS::FLI1 transcriptional targets, and antiproliferative response in vitro and in vivo [43]. To the best of our knowledge, no specific PROTAC targeting EWS::FLI1 exists. However, these data support the utility of VHL-recruiting dTAG molecules to evaluate responses associated with EWS::FLI1 loss and prove the feasibility of EWS::FLI1 targeting by PROTAC-based protein degradation [43]. This notion is further supported by evidence indicating that the control of EWS::FLI1 ubiquitination/deubiquitination is a viable strategy for controlling EWS::FLI1 protein expression. In 2016, Gierisch et al. demonstrated that ubiquitination of EWS::FLI1 at lysine 380 is critical for its proteasomal degradation [110]. Accordingly, subsequent studies have demonstrated that different E3 ubiquitin ligases are critical for EWS::FLI1 expression, including USP19 [111], SPOP [72], and TRIM8 [69]. Of note is that Seong et al. demonstrated that TRIM8 ubiquitinates and degrades EWS::FLI1, thereby regulating the stability of the chimeric protein [69]. In addition, by employing the dTAG system, the authors demonstrated that degradation of TRIM8 impairs in vitro and in vivo EWS cell growth [69]. E3 ligases are notoriously challenging drug targets. This study supports the feasibility of developing PROTACs against TRIM8 or chemical screenings to identify molecular glues that can bring TRIM8 into close proximity to EWS::FLI1 for degradation [69]. Other interactors of EWS::FLI1 susceptible to TPD include the transcription factor ETS variant 6 (ETV6). ETV6 has recently emerged as an important driver of EWS malignancy [70] as it promotes in vitro and in vivo EWS growth [70]. Mechanistically, ETV6 and EWS::FLI1 co-occupy loci genome-wide, and ETV6 blocks EWS::FLI1 protein on its target genes, promoting EWS growth [70]. For therapy, ETV6 is a challenging protein but VHL-mediated degradation of ETV6 using the dTAG system was shown as an effective strategy for mediating the proteasomal degradation of ETV6 and the inhibition of in vitro and in vivo EWS cell growth [70]. While genetically-based degradation systems have been used in experimental studies, the use of PROTAC in EWS is limited to PROTAC targeting BET proteins and PROTAC targeting CK1. BET proteins represent critical vulnerabilities in sarcomas, including EWS [71,112]. EWS::FLI1 requires BRD4 for its transcriptional activity, being part of a large complex, which includes RNA Pol II, mediator complex 1, and BRD4 [71,113]. Accordingly, BRD4 knockdown, treatment with JQ1 or PROTAC BETd attenuated EWS::ETS transcriptional signatures [71]. Notably, a strong therapeutic synergism was observed in in vitro studies combining dBET6—a CRBN-recruiting, pan-BET bromodomain degrader—and the dTAG with VHL-recruiting dTAG mediating EWS::FLI1 degradation [43]. Lenalidomide, a CK1α PROTAC that induces CK1 degradation, was successfully used to demonstrate that CK1 degradation induced EWS::FLI1 protein accumulation since CK1 phosphorilates and primes EWS::FLI1 for E3 ligase SPOP recognition and proteasomal degradation, affecting EWS cell proliferation [72]. A schematic representation of therapeutic approaches in EWS, from inhibitors to PROTAC-based strategies, is reported in Figure 2.

### 4.2. Overcoming the Resistance to BET Protein Inhibition in Osteosarcoma

**Figure 3:**
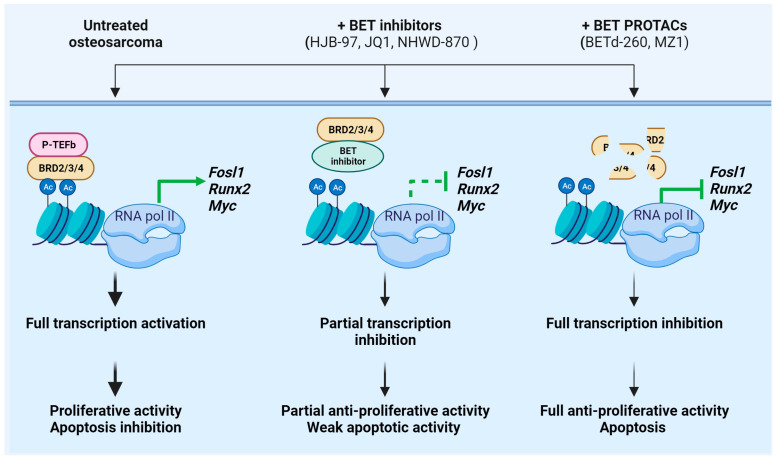
Schematic representation of the functional consequences of BET protein inhibition versus protein degradation in osteosarcoma. (**Left**): Untreated osteosarcoma cells express high levels of BET proteins BRD2, BRD3, BRD4 (BRD2/3/4), which recognize acetylated histones, thereby activating the transcription of indicated oncogenic target genes. (**Center**): Treatment with BET inhibitors partially blocks the BET proteins binding to target genes. (**Right**): BET PROTACs induce protein degradation of BRD2/3/4. Biological responses critical for gene transcription and downstream cell proliferation and apoptosis are reported.

Osteosarcoma represents the most common malignant bone tumor in children and adolescents [114]. The need for new treatment strategies in osteosarcoma is further supported by the significant short- and long-term toxicities and morbidities associated with the current treatment regimens, which include intensive multidrug therapy and surgical resection [115]. The genetics of osteosarcomas are quite complex. Osteosarcoma is characterized by elevated genomic instability and a wide range of genetic aberrations, including somatic copy-number alterations (SCNA) and structural variations (SV) but few recurrent point mutations in protein-coding genes [115]. The highly heterogeneous genetic background of osteosarcoma has significantly limited the use of targeted therapies. Accordingly, in spite of promising preclinical successes with various targeted agents, the results of those therapies in the clinic have been widely disappointing, particularly when used as monotherapy. This is the case of anti-IGF agents. For instance, an active IGF system modulates cell proliferation and migration both in vivo and in vivo as well as response to radio- and chemotherapy [112]. However, the anti-IGF-1R antibody IR3 was ineffective when used in osteosarcoma cell lines as single therapy due to autocrine compensatory mechanisms [116]. Accordingly, the anti-IGF-1R monoclonal antibody robatumumab displayed complete or partial response in only 5% of osteosarcoma patients enrolled in a phase II study [117]. Given the complexity of the osteosarcoma genome, better results were obtained in combination regimens or using multi-RTK inhibitors. The multi-RTK inhibitor regorafenib, which inhibits VEGFR1/2, c-Kit, and PDGFRβ, was tested in advanced metastatic osteosarcoma patients and displayed clinically meaningful antitumor activity, with 65% of patients receiving regorafenib not progressing at 8 weeks compared to all patients progressing in the placebo group [118]. However, it is possible that certain subsets of tumors holding unique key driver events may differentially respond to targeted therapies [119]. Using the most recent whole-genome and RNAseq techniques coupled with novel experimental models such as patient-derived xenograft (PDX) models, Sayles et al. have recently demonstrated that somatic copy-number alterations may represent novel tumor-specific dependencies in osteosarcoma [115]. The authors found copy-number somatic alteration involving *MYC*, *CCNE1*, *CDK4*, *AURKB*, and *PI3K*–*AKT*–*mTOR* signaling pathways and tested related targeted therapies according to genome-informed strategies [115]. Interestingly, the authors evaluated whether the BET inhibitor JQ1, which has been previously shown to target some MYC-driven tumors, would have a similar effect in osteosarcoma tumors carrying *MYC* copy-number somatic alteration [115]. JQ1 treatment did not determine an in vivo reduction of MYC transcript or protein levels and induced a partial inhibition of tumor growth, indicating an overall resistance to BET inhibition [115]. Accordingly, other studies indicated that suppressing BET protein action using either JQ1 as a single agent or in combination with other anticancer agents leads to modest in vitro and in vivo antiproliferative activity in osteosarcoma [115,120,121,122]. Still, osteosarcoma expresses high levels of the BET protein BRD4, and RNAseq analysis demonstrated that BET inhibitors such as HJB-97, JQ1, and NHWD-870 imposes an oncosuppressive genetic signature, affecting the expression of genes involved in cell proliferation, apoptosis, and bone destruction [120,122]. Recent findings suggest that targeting BET proteins with PROTAC degraders could have stronger therapeutic potential for osteosarcoma treatment than conventional BET inhibitors. A direct comparison between the efficacy of small molecule inhibitors JQ1 and HJB-97, and BET PROTAC BETd-260 has been recently performed [68]. The BET PROTAC BETd-260 was synthesized based on BET inhibitor HJB-97, and it recruits CRBN [123]. Of note, BETd-260 potently suppressed in vitro and in vivo cell viability of a panel of four osteosarcoma cell lines MNNG/HOS, SJSA-1, MG-63, and Saos-2 [68] with EC50 doses 1000 times lower than conventional BET inhibitors HJB-97 and JQ1 [68]. Particularly, in two xenograft osteosarcoma models, BETd-260 strongly inhibited tumor growth after a short period of administration (2–3 weeks), using low and well-tolerated doses (5 mg/kg) [68], in contrast to the modest efficacy of JQ1 alone in osteosarcoma cell line-derived xenografts [68,124]. In addition, BETd-260 triggered in vitro and in vivo apoptosis, and a strong downregulation of Bcl-2 family members and MYC [68]. Consistently with the different mechanisms of action, BET inhibitors JQ1 and HJB-97 had no effect on BET protein expression levels, while BETd-260 potently degraded BRD2/3/4 in a proteosome-dependent manner in all four tested osteosarcoma cell lines [68]. Overall, these findings suggest that complete depletion of BET proteins is more effective in treating osteosarcoma than BET inhibitors that only block histone binding [68]. Considering that osteosarcoma expresses high levels of BRD4, the use of degraders that specifically degrade BRD4 has been tested in osteosarcoma cellular models. Indeed, the use of BET PROTAC MZ1 indicated that this compound selectively induces protein degradation of BRD4 over BRD2 and BRD3 in U2OS osteosarcoma cell line [29]. Treatment with MZ1 determined a time-dependent depletion of BRD4 from the nuclei, with a complete depletion after just 3 h [29]. However, the consequences of a selective degradation of BRD4 are not fully elucidated. A schematic representation of the advantages of BET PROTACs compared to conventional BET inhibitors in the treatment of osteosarcoma is reported in Figure 3.

### 4.3. Enhancing Target Selectivity to SMARCA4/A2 in Rhabdomyosarcoma

**Figure 4:**
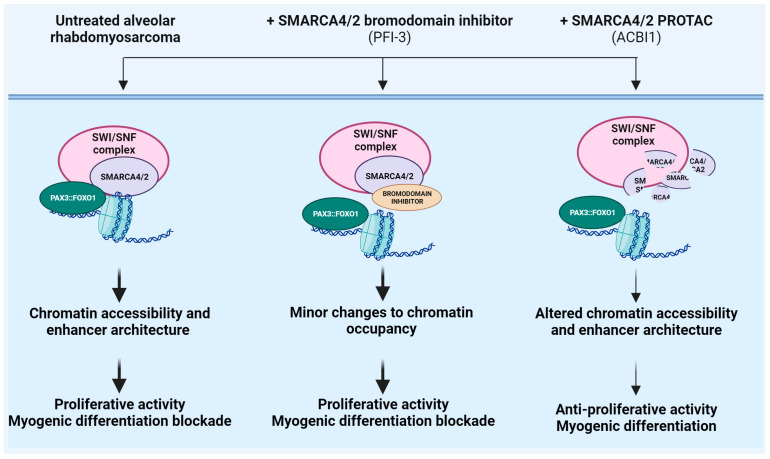
Schematic representation of the functional consequences of SMARCA2/A4 inhibition versus protein degradation in alveolar rhabdomyosarcoma (ARMS). (**Left**): Untreated ARMS cells express PAX3::FOXO1, which shares proximity on chromatin with SMARCA4, the catalytic ATPase subunit of the SWI/SNF complexes. (**Center**): Treatment with SMARCA2/4 inhibitor blocks the binding to DNA. (**Right**): Specific PROTAC induces protein degradation of SMARCA2/4. Biological responses critical for chromatin occupancy, gene transcription, and downstream cell proliferation and differentiation are reported.

Rhabdomyosarcoma (RMS) is the most common pediatric soft tissue tumor, phenocopying muscle precursors that fail to undergo terminal differentiation [125,126]. It presents with two histopathological subtypes: the alveolar and embryonal subtypes [126]. The alveolar subtype has the worst prognosis and unmet clinical need. Alveolar RMS (ARMS) is genetically characterized by recurrent chromosomal translocations t(2;13)(q35;q14) or t(1;13)(p36;q14), which fuse the DNA-binding domain of PAX3 or PAX7, respectively, to the transactivation domain of FOXO1 [127]. The resulting chimeric transcription factors PAX3::FOXO1 or PAX7::FOXO1 drive the pathogenesis of this tumor [125,126,128]. Mechanistically, PAX::FOXO1 chimeras bind and activate genomic DNA on cis-regulatory regions [127]. However, PAX3::FOXO1 and PAX7::FOXO1 have different genomic occupancy and transactivation potential [127]. In particular, PAX3::FOXO1 favors a fibroblast cellular shape associated with surface adhesion, contractility, and limited entry into the S phase [127]. In contrast, PAX7::FOXO1 induces amoeboid cellular traits, decreases entry into the M phase, and increases DNA damage [127]. Experiments with shRNA, siRNA, and CRISPR/Cas9 strategies targeting PAX::FOXO1 confirmed the strong sensitivity of fusion-positive rhabdomyosarcoma cells as demonstrated by effective inhibition of cell proliferation upon loss of the chimeric protein [129]. However, no specific inhibitor is currently available despite the fact that the direct inhibition of PAX::FOXO1 fusion proteins might represent an optimal approach for the effective inhibition of fusion-positive ARMS. The design of small molecules targeting PAX::FOXO1 is limited by the absence of targetable protein pockets as well as large protein–protein interaction interfaces [130]. Recent evidence supports the notion that targeting PAX::FOXO1 for degradation with hetero-bifunctional degraders could be a viable strategy. Using dTAG approaches in ARMS cell lines, Zhang and colleagues demonstrated that FKBP12^F36V^-PAX3::FOXO1 is susceptible to CRBN-recruiting dTAG-47 degrader [74]. Of note, treatment with dTAG-47 highlighted novel mechanisms of gene expression regulation mediated by the chimeric protein. For instance, PAX3::FOXO1 affects single elements in super-enhancers favoring RNA polymerase pause release and transcription elongation, selectively [74]. To our knowledge, no specific PAX3::FOXO1 PROTAC is currently available. However, these data support the utility of CRBN-recruiting dTAG molecules in evaluating responses associated with PAX3::FOXO1 loss and prove the feasibility of targeting PAX3::FOXO1 with PROTAC-based approaches. However, other strategies have been explored, including the targeting of pathways associated with PAX::FOXO1 stability or expression, targeting its transcriptional co-activators or targeting down-stream signaling pathways as previously reviewed [4,5]. Genome-scale CRISPR/Cas9 targeting of epigenetic genes and subsequent validation studies revealed the wild type ATP-dependent chromatin remodeling enzyme SMARCA4 as a crucial vulnerability for the survival of both human fusion-positive alveolar and fusion-negative embryonal rhabdomyosarcoma cells [129,131,132]. SMARCA4 and its paralogue SMARCA2 act as catalytic ATPase subunits of the SWI/SNF complexes, including BAF, PBAF and ncBAF, representing fundamental epigenetic regulators of gene transcription [133]. In both fusion-positive and fusion-negative cells, in vitro experiments using siRNA, shRNA, or CRISPR-mediated sgRNA *SMARCA4* knockdown approaches showed that SMARCA4 loss reduces cell proliferation, anchorage-dependent clonogenicity, and activates myogenic differentiation [129,131,132]. Mechanistically, in fusion-positive cells, SMARCA4 shares a spatial proximity with PAX3::FOXO1 on chromatin even if PAX3::FOXO1 is not incorporated into stable SWI/SNF complexes [129]. In addition, knockout of both SMARCA4 and PAX3-FOXO1 induces myogenic differentiation genes. However, the fusion protein and SWI/SNF complexes negatively regulate myogenesis through distinct mechanisms [129]. In fusion-negative cells, the over-expressed transcription factor TWIST2 favors the expression of genes related to cell growth and represses the expression of genes related to myogenesis by interacting with the chromatin remodelers SMARCA4 and CHD3 and controlling H3K27 acetylation at distal enhancers [132]. For therapeutic intervention, small molecule inhibitors targeting SMARCA2/A4 have been developed, including allosteric ATPase inhibitors (such as ATP inhibitor-1) [134], bromodomain inhibitors (such as PFI-3) [135], and the dual SMARCA4/SMARCA2 PROTAC degrader ACBI-1 [73]. From a structural standpoint, ACBI-1 incorporates a bromodomain ligand, a polyethylene glycol-based linker, and a ligand for the E3 ubiquitin ligase VHL [73]. In fusion-positive rhabdomyosarcoma, ACBI-1 demonstrated an inhibitory effect on in vitro and in vivo tumor growth, and it was associated with elongated cell morphology, which is suggestive of myogenic differentiation [129,131]. Accordingly, ACBI-1 treatment determined in vitro up-regulation of muscle differentiation genes as well as elevated expression of myosin heavy chains [129]. In fusion-negative rhabdomyosarcoma, ACBI-1 demonstrated inhibitory effects on in vitro tumor growth, although to a lesser extent, compared to fusion-positive cells [129,132]. Accordingly, fusion-negative cells did not show myogenic differentiation suggesting that SWI/SNF complex is differentially dispensable for inhibiting myogenesis in fusion-positive or fusion-negative rhabdomyosarcoma [129,132]. While ACBI-1 induced similar effects when compared to ATP inhibitor-1 in rhabdomyosarcoma cells, the SMARCA4 bromodomain inhibitor PFI-3 did not impair cell viability and did not affect myogenic differentiation of murine and human ARMS cell lines [129,135]. This is in line with findings showing that SMARCA2/A4 ATPase domains exceed bromodomains as drug targets [135]. Accordingly, ChIP experiments demonstrated that PFI-3 treatment induces minor changes to the binding of the SWI/SNF complex to genetic targets’ loci, indicating that SMARCA2/4 bromodomain inhibition is unable to displace the multisubunit SWI/SNF complex from chromatin [135]. Overall, the results indicate that targeting SMARCA4/A2 using protein degradation approaches could represent a novel therapeutic breakthrough for patients affected by ARMS [131]. A schematic representation of the therapeutic approaches, from inhibitors to degraders of SMARCA4/A2 in ARMS, is reported in Figure 4.

### 4.4. Maximizing BRD9 Blockade in Synovial Sarcoma

**Figure 5:**
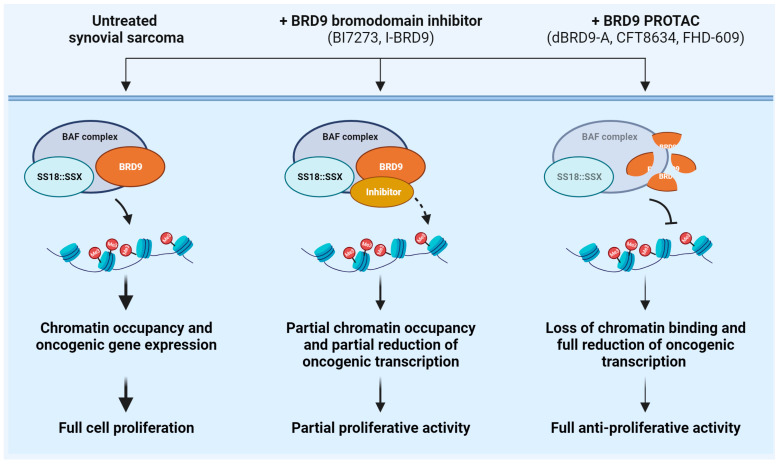
Schematic representation of the functional consequences of BRD9 inhibition versus protein degradation in synovial sarcoma. (**Left**): Untreated synovial sarcoma cells express SS18::SSX which integrates into the BAF complex along with BRD9. (**Center**): Treatment with BRD9 inhibitors partially blocks the chromatin occupancy of this complex to the DNA. (**Right**): BRD9 PROTAC induces protein degradation of BRD9. Biological responses critical for chromatin occupancy, gene transcription, and downstream cell proliferation and are reported.

Synovial sarcoma is a rare soft tissue malignancy, accounting for 8% of all soft tissue tumors. Synovial sarcoma affects patients of all ages but particularly children and young adults [136]. The genetic hallmark of this tumor is the chromosomal translocation t(X;18)(p11;q11), which causes the fusion between *SS18* (*SYT*), encoding for a component of the SWI–SNF complex, and one of the homologues *SSX* genes (*SSX1*, *SSX2*, rarely *SSX4*), encoding for a transcriptional repressor [137]. The resulting SS18::SSX fusion proteins play a crucial role in the tumorigenesis of synovial sarcoma. Accordingly, the expression of SS18::SSX in muscle progenitor cells leads to synovial sarcoma development in mice [138]. From the functional point of view, the fusion proteins integrate into the BAF (also known as SWI/SNF) and polycomb chromatin remodeling complexes epigenetically altering gene transcription [139]. Depletion of SS18::SSX fusion proteins reduces BAF complex binding at target sites and leads to the repression of fusion protein target genes, indicating that SS18::SSX represents the optimal therapeutic target in synovial sarcoma [140]. However, SS18::SSX has been so far very difficult to target for therapeutic intervention [75]. Thus, other targeted therapy-based approaches acting at epigenetic levels have been tested in preclinical and clinical settings, including enhancer of zeste homolog 2 (EZH2) and histone deacetylase (HDAC) inhibitors [141]. However, despite promising preclinical results, phase II clinical trials reported no objective response after treatment with vorinostat, panobinostat (two HDAC inhibitors; NCT00918489, NCT01136499), or tazemetostat (EZH2 inhibitor; NCT02601950). A CRISPR/Cas9-based screening approach identified Bromodomain-containing protein 9 (BRD9) as a selective functional driver of synovial sarcoma [75]. BRD9 is a non-BET bromodomain protein and a major component of the BAF complex. Proteomic analyses demonstrated that BRD9 cooperates with SS18::SSX fusion protein within BAF complexes cells and supports SS18::SSX function genome-wide in synovial sarcoma cells [75]. From the therapeutic point of view, the use of two independent BRD9 bromodomain inhibitors, I-BRD9 and BI7273, only partially inhibited synovial sarcoma cell growth and chromatin occupancy [75]. This evidence indicates that bromodomain inhibition partially ablates BRD9 function and suggests that BRD9 association with chromatin unlikely rely exclusively on bromodomain function [75]. On the contrary, better results were obtained with a targeted chemical degrader for BRD9, dBRD9-A, which hijacks BRD9 to the E3 ubiquitin ligase component CRBN [75]. Compared to the action of the small molecule BI7273, dBRD9-A treatment induced a more robust loss of BRD9 binding across the genome, thereby leading to a more effective therapeutic response than bromodomain inhibition, further supporting the notion that BRD9 can also function independently of its bromodomain [75]. Mechanistically, dBRD9-A-induced BRD9 protein degradation leads to disruption of BAF/SS18::SSX-containing complexes [75]. Treatment with dBRD9-A degrader elicited near-complete BRD9 degradation mediated by CRBN determined a robust loss of BRD9 binding across the genome, with consequent attenuation of genes associated with the oncogenic transcriptional program driven by SS18::SSX. Furthermore, BRD9 degradation induced in vitro cell cycle arrest and inhibition of in vivo tumor progression in xenograft models [75]. Interestingly, synovial sarcoma cells displayed unique sensitivity to dBRD9-A compared to other sarcoma subtypes, including Ewing sarcoma, which were not sensitive to dBRD9-A in in vitro treatments [75]. A notable exception is GISTs, which show up-regulation of BRD9 and consequent high sensitivity to dBRD9-A in in vitro models [142]. To date, two selective degraders of BRD9 are in clinical trials in patients with advanced-stage synovial sarcoma: CFT8634 (NCT05355753) and FHD-609 (NCT04965753). CFT8634 is an oral PROTAC that links BRD9 to the E3 ligase CRBN, while FHD-609 is an intravenous BRD9 degrader recruiting CRBN, and in vitro and in vivo preclinical studies have demonstrated the antitumor activity of these compounds [10,76]. Notably, two-phase I/II clinical studies are ongoing to evaluate safety, tolerability, pharmacokinetics, and pharmacodynamics of these compounds. While targeting BRD9 represents an indirect modality to block SS18::SSX, preliminary evidence shows the potential sensitivity of SS18::SSX itself to TPD. Intriguing preclinical evidence indicates that HDAC inhibitors induce proteasomal degradation of SS18::SSX proteins. In this system, the E3 ligase MULE binds to SS18::SSX and mediates SS18::SSX proteasomal degradation [143,144]. Thus, synovial sarcoma cells sustain MULE degradation by maintaining high expression of the E3 ligase MDM2, which is in turn activated by HCAD-protein-mediated acetylation [143,144]. Experimental studies using HDAC inhibitors showed that HDAC2 inhibition strongly inhibited the MDM2-MULE interaction and promoted SS18::SSX degradation. On one hand, this evidence indicates novel functions of HDAC proteins and novel effects of HDAC inhibition that can be exploited for therapy. On the other hand, these data suggest that the ubiquitin–proteasomal axis might have an impact on the expression levels of the chimeric SS18::SSX proteins, thereby supporting the need for further research to identify degraders specific for this oncogenic molecule. A schematic representation of the therapeutic approaches, from inhibitors to degraders, of BRD9 and biological consequences in ARMS is reported in Figure 5.

## 5. Conclusions

Preclinical and clinical evidence supports the utility of PROTAC-based approaches for target validation and as novel therapeutic opportunities in different types of human cancers. The development of a PROTAC is challenging due to its modular nature. In addition, research on PROTAC-based protein degradation still needs to resolve some issues associated with the limited number of recruited E3 ligases, onset of resistance mechanisms, and toxicity due to off-target/off-tissues effects. However, PROTACs hold the potential to block a wide range of oncogenic proteins, including mutated variants, transcription factors, RNA-binding proteins, and proteins acting through scaffolding modalities, thus representing a concrete opportunity to overcome the limits of the targeted therapies. After 20 years of research in the field, significant advances have been achieved in subsets of human tumors, including prostate and breast cancer, while limited studies are available in sarcomas, which represent a group of aggressive neoplasms that have poorly benefited from targeted therapies in the last decades. The majority of information obtained to date support the use of PROTAC in sarcomas, and the ability of this approach to block difficult-to-target molecules including transcription factors, E3 ligases, epigenetic readers, and chromatin enzymes. More efforts and investments are needed to bring PROTAC technology to sarcoma, but PROTACs might provide novel adjuvant strategies for the treatment of tumors with desperate need of innovative therapies.

## Figures and Tables

**Table 1:** Summary of discussed tumor types, target proteins, and related employed TPD-based approaches.

Cancer Types	Target Protein	TPD Approach	References
**Solid tumors**			
Prostate cancer	AR	ARCC-4 PROTAC	[30]
	AR Splice Variant-7	MTX-23 PROTAC	[61]
Breast cancer	Estrogen receptor	ARV-471 PROTAC	[62]
		AC682 PROTAC	
		DT2216 PROTAC	
	SGK3	SGK3-PROTAC1, HaloTag tool	[38,50]
	IGF-1R/Src	CPR3/CPR4 PROTACs	[63]
	BRD4	Ab-PROTAC 3	[64]
	BRD4	Bioorthogonal PROTAC	[65]
Lung cancer	IGF-1R/Src	CPR3/CPR4 PROTACs	[63]
	BRD4	H_2_O_2_-inducible PROTAC	[66]
	EML4-ALK fusion	dALK opto-PROTAC	[67]
Osteosarcoma	BET proteins	MZ1 PROTAC	[29]
		BETd-260 PROTAC	[68]
Ewing sarcoma	EWS::FLI1	dTAG tool	[43]
	TRIM8	dTAG tool	[69]
	ETV6	dTAG tool	[70]
	BRD4	BETd PROTAC	[71]
	CK1	CK1α PROTAC	[72]
Glioblastoma	HDAC6	J22352 PROTAC	[57]
Rhabdomyosarcoma	SMARCA4/A2	ACBI-1 PROTAC	[73]
	PAX3::FOXO1	dTAG tool	[74]
Melanoma	Mutant BRAF	SJF-0661 PROTAC	[53]
Synovial sarcoma	BRD9	dBRD9-A PROTAC	[75]
		CFT8634	[76]
		FHD-609	[76]
Cervical carcinoma	BET proteins	MZ1 PROTAC	[29]
**Hematological tumors**			
Diffuse large B-cell lymphoma	BTK	UBX-382 PROTAC	[77]
Chronic myeloid leukemia	BCR-ABL1	DMP11 PROTAC	[78]
		GMB-475 PROTAC	[79]
	LIN28	RNA-PROTAC	[80]
	STAT5	AK-2292 PROTAC	[81]
MLL-rearranged leukemia	IKZF	MS40 PROTAC	[82]
Acute myeloid leukemia	CDK2/CDK5	dTAG tool	[43]
Anaplastic large cell lymphoma	NPM-ALK fusion	dALK opto-PROTAC	[67]
